# Cerebral Venous Thrombosis: A Challenging Diagnosis; A New Nonenhanced Computed Tomography Standardized Semi-Quantitative Method

**DOI:** 10.3390/tomography8010001

**Published:** 2021-12-22

**Authors:** Andrea Romano, Maria Camilla Rossi-Espagnet, Luca Pasquini, Alberto Di Napoli, Francesco Dellepiane, Giulia Butera, Giulia Moltoni, Olga Gagliardo, Alessandro Bozzao

**Affiliations:** 1 Neuroradiology Unit, NESMOS Department, Sant’Andrea Hospital, La Sapienza University, 00189 Rome, Italy; andrea.romano@uniroma1.it (A.R.); mcamilla.rossi@opbg.net (M.C.R.-E.); luca.pasquini@uniroma1.it (L.P.); francesco.dellepiane@uniroma1.it (F.D.); giulia.butera@uniroma1.it (G.B.); giulia.moltoni@uniroma1.it (G.M.); olga.gagliardo@uniroma1.it (O.G.); alessandro.bozzao@uniroma1.it (A.B.); 2 Neuroradiology Unit, Imaging Department, Bambino Gesù Children’s Hospital, IRCCS, Piazza di Sant’Onofrio 4, 00165 Rome, Italy; 3 Neuroradiology Service, Department of Radiology, Memorial Sloan Kettering Cancer Center, New York, NY 10065, USA

**Keywords:** cerebral venous thrombosis, diagnosis, standardized method, CT, ROI based

## Abstract

Cerebral venous sinus thrombosis (CVST) on non-contrast CT (NCCT) is often challenging to detect. We retrospectively selected 41 children and 36 adults with confirmed CVST and two age-matched control groups with comparable initial symptoms. We evaluated NCCT placing four small circular ROIs in standardized regions of the cerebral dural venous system. The mean and maximum HU values were considered from each ROI, and the relative percentage variations were calculated (mean % variation and maximum % variation). We compared the highest measured value to the remaining three HU values through an ad-hoc formula based on the assumption that the thrombosed sinus has higher attenuation compared with the healthy sinuses. Percentage variations were employed to reflect how the attenuation of the thrombosed sinus deviates from the unaffected counterparts. The attenuation of the affected sinus was increased in patients with CVST, and consequently both the mean % and maximum % variations were increased. A mean % variation value of 12.97 and a maximum % variation value of 10.14 were found to be useful to distinguish patients with CVST from healthy subjects, with high sensitivity and specificity. Increased densitometric values were present in the site of venous thrombosis. A systematic, blind evaluation of the brain venous system can assist radiologists in identifying patients who need or do not need further imaging.

## 1. Introduction

Cerebral venous sinus thrombosis (CVST) is a rare but potentially life-threatening condition, with possible long-term neurological sequelae if not promptly treated. CVST is a multifactorial disease, most often affecting young adults, with female prevalence and a peak age range between 20 and 30 years [1,2,3,4,5]. With an incidence rate in the adult population of 3–4 cases per million, this condition accounts for 0.5% of cerebrovascular accidents [1]. CVST is also recognized as a cause of childhood and neonatal stroke, with an estimated mortality of 8–19% and a rate of long-term complications close to 50% [6]. 

CVST represents a diagnostic challenge due to highly variable, non-specific clinical manifestations and a wide spectrum of diagnostic imaging pitfalls [1,2,7,8,9]. Headache and seizures are the most frequent symptoms of CVST. However, the clinical presentation is often non-specific, making the diagnosis extremely difficult [10,11] and leading to diagnostic delay, which can affect patient outcome [12].

Patients admitted to the emergency department with non-specific neurological symptoms are likely to undergo an unenhanced CT scan as first line diagnostic procedure [2]. The CT scan is qualitatively evaluated by the emergency radiologist by visual inspection, with relatively low sensitivity and specificity [13,14]. When CVST is suspected, CT or MR venography are indicated. Contrast enhanced studies should only be performed if the suspicion of CVST is well founded, and the benefits outweigh the risks of further radiation exposure and administration of iodinated or gadolinium-based contrast, which may lead to brain deposition [15,16] and different types of toxicity [17,18,19].

Therefore, the availability of a quantitative spiral CT-scan diagnostic parameter, easy-to-obtain and with good sensitivity and specificity, could aid the diagnosis of CVST in an emergency setting. Several studies investigated the quantitative measurement of density of dural venous sinuses involved by thrombosis using non enhanced CT images with a retrospective approach [19,20]. This study aims to test a standardized ROI-based method to assess sinus thrombosis ignoring a priori the site of CVST.

## 2. Materials and Methods

### 2.1. Patients

Institutional review board approval approved the study. Written informed consent for CT examinations was acquired from every patient or legal tutor. All patients with a clinical suspicion of dural sinus thrombosis from January 2016 to December 2019 were retrospectively evaluated at two Institutions. All the subjects underwent plain CT and CT venography (CTV) of the brain.

### 2.2. Inclusion Criteria

- Clinical suspicion of CVST.
- No history of previous venous sinus thrombosis.
- Plain CT and CTV performed <24 h from clinical onset.

### 2.3. Exclusion Criteria

- Coexistence of other acute intracranial brain diseases.
- Presence of significant artifacts on CT images.

All the examinations were evaluated for quality assessment by four neuroradiologists with more than five years of experience.

### 2.4. CT Protocol

Non-contrast CT (NCCT) examinations were performed with helical acquisitions on two different CT scanners [16-Slice CT scanner (LightSpeed, General Electric Medical Systems, Waukesha, WI, USA) for adults and 128-slice CT scanner (Somatom Definition Flash, Siemens, Erlangen, Germany) for pediatric patients]. CTV examinations were performed on the same scanners. Venous catheters (18 gauge) were placed in the antecubital vein in all patients. Contrast agent (70 mL) with an iodine concentration of 300 mg/mL was injected and imaging was performed with delay of 35 s, a flow rate between 3 and 5 mL/s was chosen.

Maximum intensity projections (MIPs) were created for the entire region of interest in axial, coronal and sagittal orientations with an increment of 3 mm.

### 2.5. Image Analysis

Plain CT images were evaluated using a ROI-based analysis by two observers with more than 5 years of experience in Neuroradiology, blinded to the final and to the results of the CTV. All the obtained CT images were reconstructed on the bi-commissural plane to reproduce the same anatomic condition for ROIs placement. 

**Figure 1:**
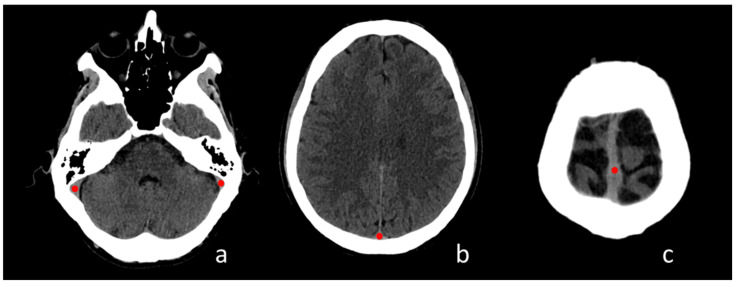
Regions of Interest placement in four standardized regions of the cerebral venous system; (**a**) transverse-sigmoid sinus passage; (**b**) posterior third of the superior sagittal sinus (SSS); (**c**) middle third of the SSS.

Four circular ROIs (diameter: 5 mm in the adult group and 3 mm in the pediatric group) were placed in predetermined locations: left and right transverse-sigmoid sinus passage, posterior third and middle third of the superior sagittal sinus (SSS) at the vertex following a standard reference (Figure 1). In the case of transverse sinus asymmetry (due to hypoplasia), the ROI was adapted to the sinus diameter visible at CT scan. 

Mean and maximum HU density values from each of the four circular ROIs placed inside the sinuses were obtained. The % relative difference between the value with higher density and the other three was calculated with mean and maximum HU values, using the following formula:

$$100-\left\{\frac{\left(ROI+ROI+ROI\right)/3\left(with\;lower\;HU\;max\;or\;mean\;values\right)}{ROI\;with\;higher\;HU\;max\;or\;mean\;values}\times 100\right\}$$

### 2.6. Statistical Analysis

The statistical evaluation was performed with SPSS software (version 20.0, Chicago, IL, USA). A Two-Sample *t*-test was obtained to compare mean and maximum HU% relative difference between patients with and without CVST (level of statistical significance: *p* < 0.05), also between adult and pediatric subgroups, respectively.

Empiric receiver characteristics (ROC) curves of relative HU percent value (using mean and maximum HU values, respectively) were obtained and compared to determine the best cutoff value able to distinguish between patients with CVST and not. The Inter-rater reliability was calculated by means of Cohen’s K test.

## 3. Results

### 3.1. Subject Population

Ninty-eight patients with suspected CVST were evaluated. Among these, 1 patient was excluded because of previous CVST; 10 patients were excluded because of subtle partial venous thrombosis associated with skull fracture; 6 patients were excluded because of other concomitant brain pathology (one with epidural hematoma at the level of a superficial venous sinus, four with acute lymphoblastic leukemia with intracranial localization, and one with intracranial meningitis/encephalitis); 3 subjects were excluded because of significant motion artifacts on brain scans; and 1 patient was excluded because of venous thrombosis secondary to a neurosurgical complication.

A total of 77 patients (33 F and 44 M) with a mean age of 38.7 years (age range: 1 month–75 years) were included in the study. Of these, 29 patients (14 F and 15 M) with a mean age of years 36.7 years (age range: 1 month–69) had confirmations of CVST with CTV. Only nine female patients declared the use of contraceptive therapy; no other risk factors were reported.

Among patients with CVST, five showed involvement of three venous sinuses, nine patients demonstrated involvement of two venous sinuses, and 14 patients had only one sinus affected. 

### 3.2. Data Analysis

**Figure 2:**
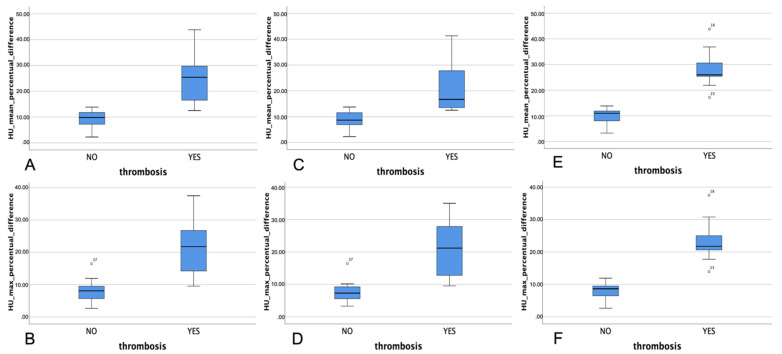
Box-plots showing the difference in Hounsfield Units (HU) mean percentual difference (**A**,**C**,**E**) and maximum percentual difference (**B**,**D**,**F**) respectively in all patients (**A**,**B**), adult (**C**,**D**) and pediatric (**E**,**F**) patients with and without thrombosis. Variation in patients with and without thrombosis; (**B**) box-plots showing the difference in HU maximum percentual variation in patients with and without thrombosis; (**C**) receiver operating characteristic (ROC) curve with cut-off for HU mean and HU maximum differences variations.

In all cases, the most hyperdense sinus sign correlated with the side of the sinus thrombosis. A significant difference was appreciable comparing all parameters evaluated (mean HU% relative difference value, maximum HU% relative difference value) between patients with and without CVST (Figure 2A,B); similar results were obtained comparing adult and pediatric subgroups with and without CVST (Figure 2C–F) (Table 1).

**Figure 3:**
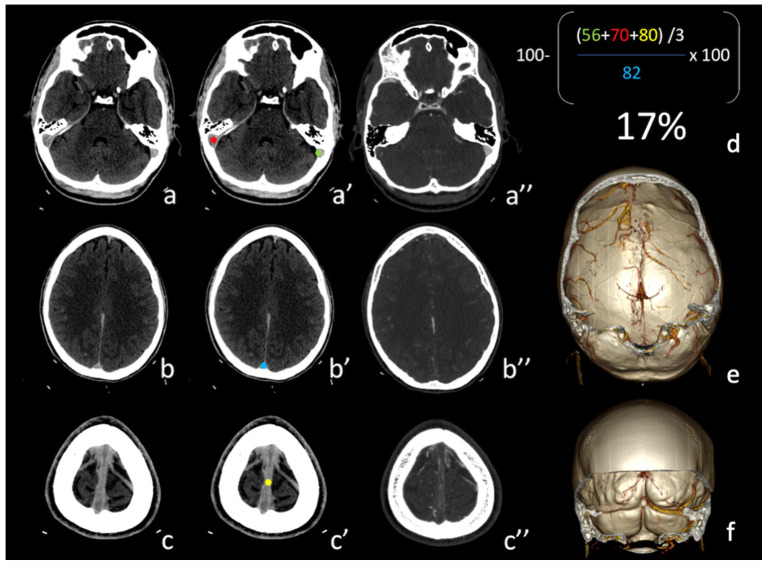
(**a**–**c**) Non-contrast CT (NCCT) images of a patient with superior sagittal sinus and right transverse sinus thrombosis; (**a’**–**c’**) example of the proposed method applied to the patient’s cerebral venous system with the proposed formula (**d**) indicative of venous thrombosis; (**a”**–**c”**,**e**,**f**) CT angiography that confirms superior sagittal sinus and right transverse sinus thrombosis.

Including all patients, according to ROC analysis (Figure 2C) the area under curve was 98% for both mean minimum and maximum densities, 97% for adult group and 100% for pediatric group. The optimized cut-off to distinguish patients with and without CVST are reported in Table 2. A representative case is shown in Figure 3.

We can speculate that a mean relative difference value lower than 10% among the average of minimum and maximum densities represents an indicative threshold for the absence of thrombosis (specificity of 100%). We found a good interclass correlation coefficient for percent values of 0.831.

## 4. Discussion

The aim of this study was to provide an easy-to-apply standardized method for the diagnosis of CVST. In our experience, the use of the proposed formula (based on the ratio between the HU of the most hyperdense sinus over the others) assessed the risk of thrombosis with high accuracy. A mean % relative difference value cut-off less than 10 excluded a sinus thrombosis with 100% specificity. If this observation is confirmed by larger series, this could lower the number of contrast enhanced studies for this diagnosis (CTV).

Plain CT is the first imaging study in the emergency setting in most institutions [21]; visual assessment of dural sinuses or quantitative evaluation of vessel density compatible with thrombosis are reported in literature [21]. Typically, the mean attenuation value of dural sinuses is 50 HU, with a normal range between 35 and 65 HU. Blood density is influenced by hemoglobin (HGB) levels, and there is a moderate correlation between HGB levels and attenuation measurements of dural sinuses. [22]. 

Many studies underlined the utility of quantitative methods (alone or with correlation to the hematocrit status) also calculating different density ratios with retrospective evaluations. Hyper density values cut-offs ranging from 58 and 89 HU lead to a sensitivity of 68–100% and a specificity of 81–100% in previous studies [22,23,24,25].

Black et al. first proved that basal attenuation values ≥ 70 HU highly correlate with CVST. They suggested the potential usefulness of HU values and H:H ratio (H:H = Hounsfield unit-to-hematocrit ratio) in the diagnosis of CVST, finding a correlation between hemoconcentration and attenuation of cerebral venous sinuses on non-contrast CT. They recommended an H:H ratio greater than or equal to 2 as the cut-off value to discriminate patients with CVST [22]. 

Buyck et al., suggested a threshold of 62 HU to evaluate CVST, confirming that the measurement of sinus density and H:H ratio on unenhanced brain CT, may increase the confidence of the radiologist, and clarify the need for confirmatory CTV or MR imaging studies [23]. 

Previous studies supported the idea of the quantitative evaluation of HU values being preferable to the H:H ratio, which may lead to a higher number of false positives [23]. Other studies used arterial density as a reference. Tayyebi et al. and Alsafi et al. compared density values of venous sinuses in patients with and without thrombosis before and after normalization to the average HU of the basilar artery and ICAs [25,26].

Besachio et al. reported that in addition to HU values and H:H ratio, a venous-arterial difference value greater than 15 correlated to CVST [27]. A similar method was employed by Zaheer et al. with a ROI-based measurement of a superficial dural sinuses HU density. Densitometric values of target sinuses were compared before and after normalization to average HU of the basilar artery, internal carotid arteries, temporal and frontal lobes [28].

The advantage of our proposed method is to evaluate the dural sinus density on non-enhanced CT images and the presence of a CVST, without a retrospective evaluation of them after angiographic examination, as mentioned before. Concerning pediatric populations, De la Vega Muns et al. in a ROI-based analysis described that a maximum value 58 HU with 1.4 HU:HCT ratio was found to be significantly related to CVST [10].

Two more recent studies, a multicenter research [29] and a meta-analysis [30], reported some limitations of retrospective quantitative evaluations of CVST. Buyck et al. assessed that the visual analysis of plain CT scans was highly specific but only moderately sensitive for CVST detection, and the semi-quantitative measure threshold employed in the study did not achieve the sensitivity and specificity targets [29]. Xu et al. reported that the visual assessments of direct (cerebral damages) and indirect CT signs (cord signs and hyperdense sinus) of CVST appeared to have a better performance compared to quantitative measurements, although the results of CT quantitative analysis led to good diagnostic accuracy [30].

Similar conclusions were achieved by Roland et al. These authors reported that unenhanced brain CT had a sensitivity of 73% to detect CVST when both primary and secondary signs of thrombosis were considered. The authors concluded that density measurements do not significantly improve the sensitivity of unenhanced CT, recommending to assess the need for additional imaging based on clinical presentation, predisposing factors and unenhanced CT [13].

Buyck et al. calculated the density ratio placing a circular ROI in the affected venous sinus and contralateral unaffected counterpart [29]. This method did not require knowledge of the hematocrit, as in our study, and is, therefore, available in the emergency setting, where laboratory testing may delay the diagnosis. The authors did not reach high enough sensitivity to confidently rule out CVST, since the marker was difficult to obtain when bilateral thrombosis occurred or when only the superior sagittal sinus was involved. The authors concluded with re-affirming current guidelines that require additional imaging with CTV or MRV when a CVST is suspected.

To overcome all the potential limitations discussed above, we used a different approach, independent from subjective visual inspection, by calculating the ratio between the sinus with the highest density value and the other sinuses. Using the proposed formula, which considers all the dural sinuses (see the Methods section), we were able to identify the patients with CVST and the specific site of thrombosis. We found good cut-off values to assess these pathological conditions for all patient categories (all patients, adult and pediatric sub-groups). Based on the proposed cut-off, patients with above-threshold percentual variation might require more advanced neuroimaging, such as CTV.

We speculate that a mean relative difference value lower than 10% among the average of minimum and maximum densities represents an indicative threshold for the absence of thrombosis (specificity of 100%). This result could be used to stratify patients, avoiding futile CT angiography, reducing contrast administration and radiation exposure.

This study has several limitations. Over-estimation and false positive results could occur using the proposed formula or the conventional quantitative approach. The dural sinuses are immediately adjacent to the inner table of the skull, which is frequently grooved by these venous structures. Atretic or hypoplastic venous sinuses are a common diagnostic pitfall in neuroimaging.

For example, in almost half of the patients, the transverse sinus is asymmetric, and in 20% of patients, either side may be partially or completely absent [31]; consequently, a false ROI positioning is possible. To avoid potential mistakes, we chose the transverse-sigmoid passage as a region of interest (which was always possible to assess). A venous thrombosis that involves all dural sinuses could represent another diagnostic pitfall. Although this condition is unusual, especially for children, it should not be under evaluated.

Our method can underestimate the number of patients affected by partial CVST. This aspect represents another limitation of the study, in particular in the pediatric group, where this pathologic condition is more frequent. For this reason, we decided to exclude patients with other brain diseases (such as fractures or infectious diseases) that might lead to CVST and need, by definition, CTV in the case of suspected thrombosis. The number of subjects is another limitation of this study. Although 29 atients with CVST and 48 without disease were considered, larger studies are needed to confirm our results.

## 5. Conclusions

We demonstrated that the proposed method represents a good procedure to stratify patients with suspected CVST and, if necessary, to guide them to further imaging, avoiding futile CT angiography and reducing contrast administration and radiation exposure. 

## Figures and Tables

**Table 1:** Results with mean Hounsfield unit percentage (HU%) and max HU% in patients with and without cerebral venous sinus thrombosis (CVST). The second and third rows show results in the adult and pediatric subgroups, respectively.

		Thrombosis	Mean	Standard Deviation	*p*
CVST patients vs. no CVST patients	mean HU% relative difference	no	9.4	3	
		yes	24.1	8.9	<0.001
	max HU% relative difference	no	7.8	2.6	
		yes	21.2	7.8	<0.001
CVST adult patients vs. no CVST adult patients	mean HU% relative difference	no	8.6	3.4	
		yes	21.5	9.1	<0.001
	max HU% relative difference	no	7.6	3.1	
		yes	19.9	8.2	<0.001
CVST pediatric patients vs. no CVST pediatric patients	mean HU% relative difference	no	9.8	2.7	
		yes	28.1	7.2	<0.001
	max HU% relative difference	no	8	2.3	
		yes	23.3	6.3	<0.001

**Table 2:** Optimized cut-off for distinguish patients with and without cerebral venous sinus thrombosis. The second and third rows show analysis in the adult and pediatric subgroups, respectively. PPV = positive predictive value; and NPV = negative predictive value.

	Mean % Relative Differences					Max % Relative Differences				
	Cut-Off	Sensitivity	Specificity	PPV	NPV	Cut-Off	Sensitivity	Specificity	PPV	NPV
all patients	12.97	96%	0.92	92%	89%	10.14	96%	90%	88%	86%
adult group	12.97	94%	95%	92%	90%	10.14	94%	95%	92%	90%
pediatric group	15.52	100%	100%	100%	100%	12.91	100%	100%	100%	100%

## Data Availability

Data are available upon request to the authors.
